# Clinical evaluation of magnetic resonance imaging in coronary heart disease: The CE-MARC study

**DOI:** 10.1186/1745-6215-10-62

**Published:** 2009-07-29

**Authors:** John P Greenwood, Neil Maredia, Aleksandra Radjenovic, Julia M Brown, Jane Nixon, Amanda J Farrin, Catherine Dickinson, John F Younger, John P Ridgway, Mark Sculpher, Stephen G Ball, Sven Plein

**Affiliations:** 1 Division of Cardiovascular and Neuronal Remodelling, Leeds Institute of Genetics, Health and Therapeutics, University of Leeds, G-Floor, Jubilee Wing, Leeds General Infirmary, Great George Street, Leeds, LS1 3EX, UK; 2 Division of Medical Physics, University of Leeds, Leeds, LS2 9JT, UK; 3 Clinical Trials Research Unit, University of Leeds, Clinical Trials Research House, 71-75 Clarendon Rd, Leeds, LS2 9PH, UK; 4 Department of Nuclear Cardiology, Leeds Teaching Hospitals NHS Trust, Leeds General Infirmary, Great George Street, Leeds, LS1 3EX, UK; 5 Department of Medical Physics, Leeds Teaching Hospitals NHS Trust, Leeds, LS2 9JT, UK; 6 Centre for Health Economics, University of York, Heslington, York, YO10 5DD, UK

## Abstract

**Background:**

Several investigations are currently available to establish the diagnosis of coronary heart disease (CHD). Of these, cardiovascular magnetic resonance (CMR) offers the greatest information from a single test, allowing the assessment of myocardial function, perfusion, viability and coronary artery anatomy. However, data from large scale studies that prospectively evaluate the diagnostic accuracy of multi-parametric CMR for the detection of CHD in unselected populations are lacking, and there are few data on the performance of CMR compared with current diagnostic tests, its prognostic value and cost-effectiveness.

**Methods/design:**

This is a prospective diagnostic accuracy cohort study of 750 patients referred to a cardiologist with suspected CHD. Exercise tolerance testing (ETT) will be preformed if patients are physically able. Recruited patients will then undergo CMR and single photon emission tomography (SPECT) followed in all patients by invasive X-ray coronary angiography. The order of the CMR and SPECT tests will be randomised. The CMR study will comprise rest and adenosine stress perfusion, cine imaging, late gadolinium enhancement and whole-heart MR coronary angiography. SPECT will use a gated stress/rest protocol. The primary objective of the study is to determine the diagnostic accuracy of CMR in detecting significant coronary stenosis, as defined by X-ray coronary angiography. Secondary objectives include an assessment of the prognostic value of CMR imaging, a comparison of its diagnostic accuracy against SPECT and ETT, and an assessment of cost-effectiveness.

**Discussion:**

The CE-MARC study is a prospective, diagnostic accuracy cohort study of 750 patients assessing the performance of a multi-parametric CMR study in detecting CHD using invasive X-ray coronary angiography as the reference standard and comparing it with ETT and SPECT.

**Trial Registration:**

Current Controlled Trials ISRCTN77246133

## Background

Coronary heart disease (CHD) is a major cause of morbidity and mortality in the UK, affecting approximately 2.6 million people [1]. In a typical hospital setting, a variety of investigations may be used to diagnose CHD, risk stratify patients and plan their clinical management. The highest risk patients may proceed directly to X-ray coronary angiography, but the vast majority of patients at low to intermediate risk undergo non-invasive assessment in the form of exercise tolerance testing (ETT), stress echocardiography or single photon emission computed tomography (SPECT), in order to identify those most likely to require coronary revascularisation. The diagnostic process commonly involves a multiple-test strategy, either due to limitations in the performance of any individual test (e.g. ~30% of patients may be physically unable to perform an adequate ETT) or limitations in the test's diagnostic accuracy. For example, the reported sensitivity and specificity of ETT for angiography-proven CHD was 68% and 77% respectively from a large meta-analysis where the prevalence of CHD was high at over 60% [2]. SPECT provides greater diagnostic accuracy than ETT, 86% and 74% sensitivity and specificity respectively, compared to X-ray angiography for the diagnosis of CHD [3], being typically performed in patients with equivocal ETT results, those who cannot exercise or those who have fixed resting ECG abnormalities such as left bundle branch block [3].

The need for a reliable screening tool to select patients for invasive X-ray coronary angiography stems from the risks associated with exposure to ionising radiation and the small but significant morbidity and mortality associated with the procedure (0.11% risk of procedural death, 0.05% myocardial infarction, 0.07% stroke) [4]. The issue of radiation is often overlooked, but the risk of developing a solid tumour has been estimated at 1 in 2500 diagnostic coronary angiographic procedures [5,6]. In 2006, approximately 216,000 X-ray coronary angiograms, 74,000 percutaneous coronary interventions [7] and 20,000 coronary artery bypass graft operations were performed in the UK [8]. It is therefore evident that a significant proportion of patients undergoing coronary angiography do not subsequently require revascularisation and hence the X-ray coronary angiogram is performed only for diagnostic purposes. Cardiac Magnetic Resonance (CMR) might reliably identify those patients who actually require revascularisation and thereby reduce the need for unnecessary invasive investigation in the remainder.

### CMR imaging

**Figure 1:**
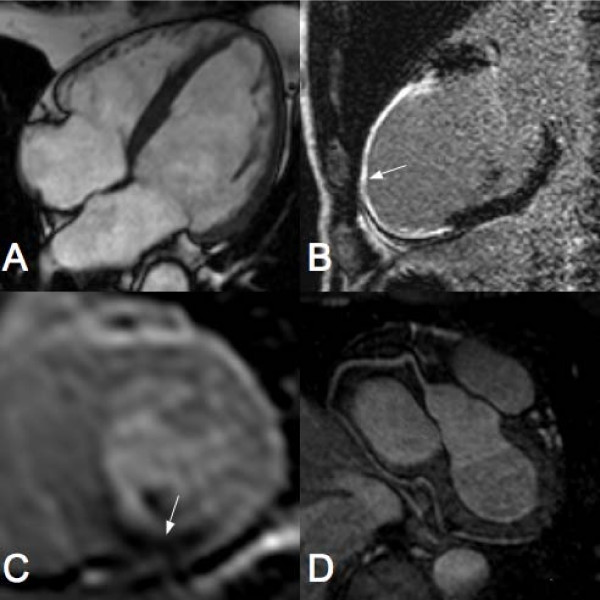
**Sample cardiac MR images**. A) Standard four chamber SSFP view of the heart LV showing both atria and ventricles. B) Vertical long axis late gadolinium enhancement view of the LV showing infarction of the anteroapical wall (arrow). C) Short axis view of the LV showing a perfusion defect in the inferior wall (arrow). D) Typical MR angiogram of a right coronary artery.

CMR is the most accurate and reproducible technique for morphological imaging of the heart because of its outstanding image resolution and tissue contrast. In addition to providing detailed anatomical information, CMR can assess myocardial function, perfusion, tissue viability, coronary artery anatomy and coronary blood flow with accuracy similar or superior to that provided by other established tests (Fig. 1) [9-16]. Furthermore, CMR is safe, non-invasive and does not expose patients to ionising radiation.

A major strength of CMR is that ventricular function, tissue viability, myocardial perfusion and coronary anatomy can be assessed in a single comprehensive study [15,16]. To obtain equivalent information by current clinically validated techniques would require a combination of echocardiography, ETT and/or SPECT as well as invasive or CT coronary angiography. CMR may therefore more accurately and more cost effectively identify those patients with suspected CHD who require coronary revascularisation than the current 'battery of tests'. It has been shown that combining the four components function, viability, perfusion and coronary anatomy into a single CMR examination improves the sensitivity for the diagnosis of significant CHD over and above that for any individual component, with a combined sensitivity and specificity for the comprehensive analysis of 96% and 83% respectively [16]. These findings have been corroborated by a recent study by Klem *et al*, which found the combination of CMR perfusion and late gadolinium enhancement CMR to be superior in detecting coronary artery disease than CMR perfusion alone [17].

The recent MR-IMPACT study was the first multi-centre study comparing the diagnostic ability of CMR and of SPECT in comparison with X-ray angiography in 241 patients and showed equal performance of the two tests [18]. An important limitation of this and previous CMR publications as well as most of the SPECT literature, is that the recruited patients were already listed for coronary angiography or that analysis was retrospective, introducing an important selection bias into these studies.

### Aims and objectives

The primary objective of this study is to assess the diagnostic accuracy of CMR in detecting significant CHD compared to the current 'reference standard' X-ray angiography. Secondary objectives will be to a) compare the diagnostic accuracy of CMR in detecting significant CHD against the current standard clinical investigations of ETT and SPECT; b) assess the prognostic value of CMR in predicting time to a major adverse cardiovascular event and c) to evaluate the cost effectiveness of CMR in a diagnostic strategy for the systematic investigation of patients with suspected CHD.

## Methods

### Study design

This is a prospective, diagnostic accuracy cohort study of patients referred to cardiologists for the further evaluation of symptoms thought to be angina pectoris. All recruited patients will undergo SPECT, CMR, ETT (when physically capable) and X-ray angiography as part of this study. Patients will be recruited from Leeds Teaching Hospitals NHS Trust, Leeds, UK and Pinderfields Hospital, Wakefield, UK. All SPECT and CMR scans will be undertaken at Leeds General Infirmary. The study will be performed in accordance with the Declaration of Helsinki (October 2000), with all patients providing informed written consent. The study protocol and other relevant documentation has been approved by the Leeds (West) Research Ethics Committee (REC). Patients will then be randomised, via an automated 24-hour randomisation system, to the order in which they undergo SPECT and CMR imaging. Stratified permuted block randomisation will be used to ensure that the groups are well balanced for age (<65, ≥65) and gender. Finally all patients will undergo X-ray coronary angiography, regardless of the treating physician's chosen clinical pathway, within 4 weeks of randomisation, which will act as the reference standard.

Statistical analyses will be carried out by the Clinical Trials Research Unit (CTRU), University of Leeds, UK and the Centre for Health Economics, University of York, UK. The study population will be followed up prospectively for a minimum of 3 years to establish the prognostic value of CMR in predicting long-term major cardiovascular events.

### Eligibility

Inclusion criteria for the study are stable symptoms thought to be angina pectoris, at least one cardiovascular risk factor (smoking, family history of premature cardiovascular disease, arterial hypertension, hyperlipidaemia, diabetes mellitus), body weight less than 110 kg, suitability for coronary revascularisation if required and sinus rhythm.

Exclusion criteria for the study are previous coronary artery bypass surgery (but not percutaneous coronary intervention), evidence of crescendo angina or acute coronary syndrome, contraindication to CMR imaging (e.g. pacemaker, intra-orbital metallic debris, intracranial clips) or adenosine infusion (regular adenosine antagonist medication, reversible airways disease, second or third degree atrio-ventricular heart block, sino-atrial disease), pregnancy, known adverse reaction to Gadolinium-based contrast agents, inability to lie supine for 60 minutes and chronic renal failure (estimated glomerular filtration rate ≤ 30 ml/min).

### Recruitment and data collection

Patients will be identified from consecutive referrals to rapid access chest pain and general cardiology clinics in the Leeds and Wakefield area, which has a combined population of approximately one million people. Patients will be reviewed by a clinician and will have an ETT if they are physically able. Patients who are deemed suitable and who are proceeding for further investigations will be offered the chance to participate in the study.

An anonymised log of all patients screened for eligibility who are not recruited either because they are ineligible or because they decline participation will be kept. If a patient is enrolled in the study, information on risk factors including age, gender, cholesterol, blood pressure, smoking and diabetes, will be collected. After enrolment, the treating clinician will be asked to make a provisional diagnosis (definite angina, probable angina, possible angina or atypical symptoms) and indicate what their planned sequence of investigations (with options of ETT, SPECT and X-ray angiography or none) for this individual patient would have been, had they not participated in the study. This clinical intention record will allow assessment as to whether the use of CMR, or SPECT, impacts on the outcome for individual patients. It will not affect the study investigations and every patient will undergo all three study investigations in the order prescribed for the study.

### Investigation details

#### CMR

**Figure 2:**
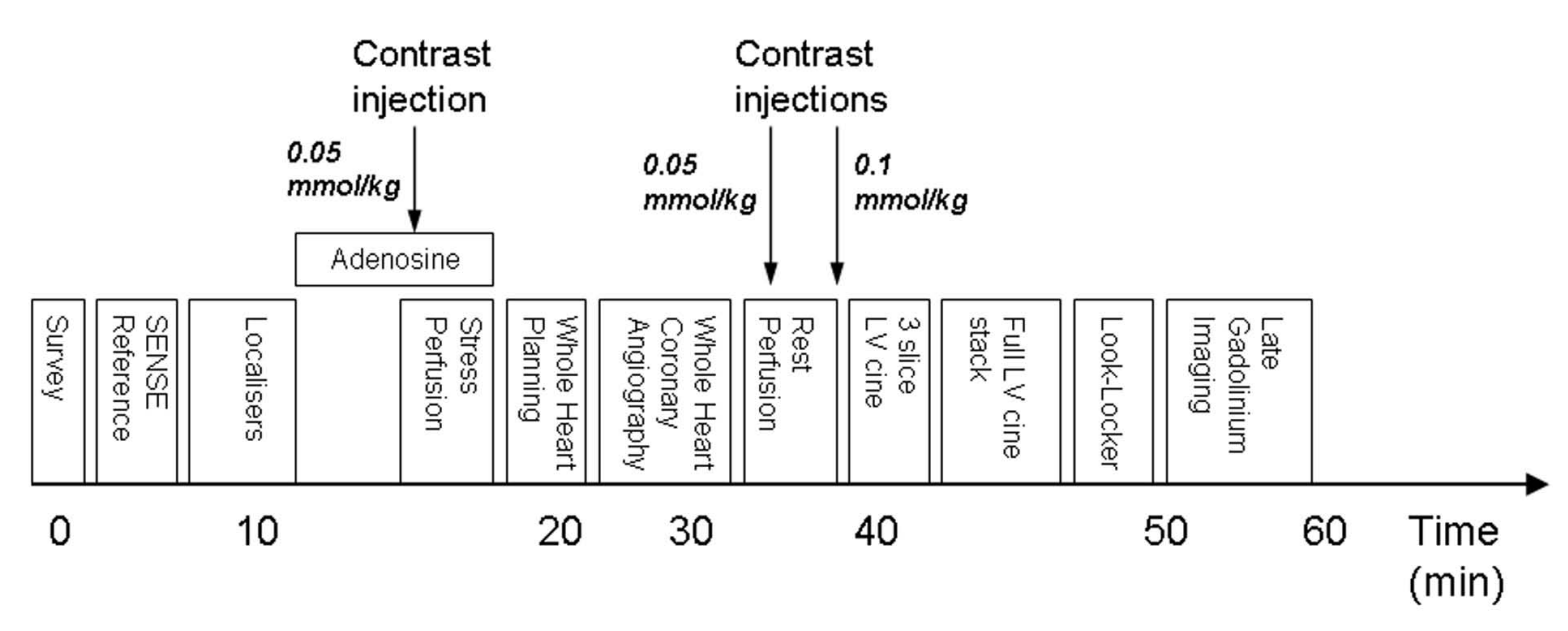
**CE-MARC cardiac magnetic resonance protocol**. The protocol commences with a low-resolution survey scan and localisers. Intravenous adenosine is then administered for approximately 4 minutes at 140 mcg/kg/min, following which first pass stress perfusion imaging is undertaken after the injection of 0.05 mmol/kg dimeglumine gadopentetate. Three dimensional whole heart MR coronary angiography follows the low resolution coronary survey and free-breathing 4 chamber cine (used to assess slice coverage and diastolic coronary rest period respectively). Rest perfusion imaging is undertaken a minimum of 15 minutes following stress perfusion, with a further injection of 0.05 mmol/kg dimeglumine gadopentetate. A final injection of 0.1 mmol/kg dimeglumine gadopentetate is given following this sequence, bringing the overall gadolinium dose to 0.2 mmol/kg. Resting left ventricular function is then assessed, initially for three slices planned identically to the perfusion slices, and then for the entire left ventricle using contiguous slices. A modified Look-Locker inversion time scout is performed prior to late gadolinium enhancement imaging in short axis, vertical long axis and horizontal long axis orientations. Times indicated on the diagram are approximate and sequence blocks are not drawn to scale.

CMR studies will be carried out on a dedicated cardiac research scanner (1.5 Tesla Intera CV, Philips, Best, The Netherlands) equipped with 'Master' gradients (30 mT/m peak gradients and 150 mT/m/ms slew rate) based at the Leeds General Infirmary, Leeds, UK. The CMR signals are received by a 5-element cardiac phased-array coil and ECG gating and triggering will be performed by the vectorcardiographic method. The total scan duration is approximately one hour and will comprise (Fig. 2):

1. Low resolution survey and sensitivity encoding (SENSE) reference scans performed prior to the cardiac localiser scans which define short axis, vertical long axis and horizontal long axis acquired with a balanced steady state free precession (SSFP), single slice, breath-hold pulse sequence. Pulse sequence parameters: echo time (TE) 1.6 ms, repetition time (TR) 3.2 ms, slice thickness 8 mm, matrix 192 × 192, field of view 320–400 mm according to patient size, SENSE factor 1.7 to 2.0, 30–50 phases per cardiac cycle.

2. Stress myocardial perfusion study. A T1-weighted saturation-recovery segmented k-space gradient echo pulse sequence combined with SENSE will be used to assess first pass myocardial perfusion in 3 short axis slices, positioned according to the "3 of 5" technique [19]. Pulse sequence parameters: TE 1.0 ms, TR 2.7 ms, flip angle 15°, single saturation pre-pulse per R-R interval shared over three slices, SENSE factor 2, matrix 144 × 144, field of view 320–460 mm, slice thickness 10 mm. For stress perfusion imaging intravenous adenosine will be administered at a dose of 140 μg/kg/min. The patient's blood pressure will be recorded every two minutes and the heart rhythm monitored on the vector-ECG. The perfusion study will commence approximately 4 minutes into the adenosine infusion. A bolus intravenous injection of 0.05 mmol/kg dimeglumine gadopentetate (Magnevist^®^, Schering AG, West Sussex, UK) followed by a 15 ml saline flush will be delivered through an arm vein at 5 ml/s using a power injector (Spectris^®^, Medrad, Pittsburgh, Pennsylvania), while the patient holds their breath in end-expiration.

3. Coronary MR Angiography. A low-resolution coronary survey scan will be performed during free breathing, using a respiratory navigator. Timing of the diastolic coronary rest period is estimated from the four-chamber free breathing cine scan. Three dimensional, whole heart coronary MR angiography is acquired using a balanced SSFP sequence and a respiratory navigator to compensate for respiratory motion during free breathing. Pulse sequence parameters: TE 2.3 ms, TR 4.6 ms, flip angle 100°, T2 and fat saturation pre-pulses, SENSE factor 1.7, duration of acquisition up to 120 ms per R-R interval (determined by length of diastolic rest period), matrix 304 × 304, field of view 320–460 mm, slice thickness 0.9 mm, 100–120 slices as required.

4. Resting myocardial perfusion study. Pulse sequence, slice positioning, and injection characteristics identical to the stress perfusion scan.

5. Additional intravenous injection of dimeglumine gadopentetate (0.1 mmol/kg), given within 60 seconds of the rest perfusion scan, in preparation for late gadolinium enhanced CMR.

6. Resting wall-motion. Contiguous cine stack encompassing the entire left ventricle in 10–12 slices (depending on left ventricular long axis length). Three additional slices, with identical slice positioning to the perfusion sequence will also be acquired. Pulse sequence parameters: balanced SSFP, TE 1.7 ms, TR 3.5 ms, flip angle 60°, SENSE factor 2, matrix 192 × 192, field of view 320–460 mm, slice thickness 10 mm, at least 20 phases per cardiac cycle, 1–2 slices per breath-hold.

7. Late gadolinium enhanced CMR performed between 10 and 20 minutes after step 5. The optimal inversion time to null signal from normal myocardium will be determined using a modified Look-Locker approach [20]. Subsequently, a T1-weighted, segmented inversion-recovery gradient echo sequence will be used to acquire a contiguous stack of short axis slices covering the entire left ventricle. Pulse sequence parameters: non-selective 180° pre-pulse, TE 1.9 ms, TR 4.9 ms, flip angle 15°, inversion time adjusted individually according to the Look-Locker scan, 10–12 short axis slices, single slice per breath-hold, matrix 240 × 240, field of view 320–460 mm as per patient size. Further slices will be acquired in the vertical and horizontal long axis orientations (at least one slice per orientation, more if clinically indicated.)

#### ETT

Exercise tolerance testing will be performed if the patient is able to exercise appropriately. This will take place at the recruiting hospital using standard Bruce protocol in most cases, although alternate protocols can be used if required. The following data will be recorded: Resting heart rate and BP, peak exercise stress heart rate and exercise duration, achievement of 85% age predicted heart rate and total workload (Metabolic Equivalents, METS), reason for test termination including reproduction of clinical symptoms, degree of ST segment shift, presence of arrhythmia and heart rate recovery at one minute.

#### SPECT

SPECT radionuclide imaging will be carried out a dedicated cardiac gamma camera (MEDISO Cardio-C, Budapest, Hungary) based at the Leeds General Infirmary, Leeds, UK. Patients will undergo a two day scanning protocol using the radioisotope tracer 99mTc tetrofosmin (Myoview), standard dose of 400 MBq for each examination, adjusted to weight to a maximum of 600 MBq per examination. Stress and rest ECG gated SPECT images will be acquired with the patients in the supine position with a total of 64 projections, 3 degree interval, every 40 seconds over a 180 degree orbit. At each projection 8 ECG gated frames per cardiac cycle will be acquired. A matrix size of 64 × 64 will be used. Trans-axial stress and rest slices of 6 mm thickness will be reconstructed with a Butterworth scattered back-projection filter, with a cuttoff frequency of 0.4 Nyquist and an order of 6. Transaxial slices will be reorientated to the cardiac axes for analysis. Data will be processed using QGS software (Cedars-Sinai Medical Center, USA) to calculate end diastolic and end systolic volumes and wall motion scores. QPS (Cedars-Sinai Medical Center, USA) will be used to for semiquantitative analysis of the perfusion data including summed stress and rest scores. The stress imaging protocol will be performed using intravenous adenosine (140 mcg/kg/min) for 4 minutes followed by isotope injection so that the perfusion techniques of SPECT and CMR are directly comparable.

#### X-ray Angiography

All patients in this study will undergo invasive X-ray coronary angiography by a cardiologist who will be blinded to SPECT and CMR results. Patient management decisions will be made independently of study participation. The results of CMR will not be routinely available to clinicians. The SPECT results will be made available at the request of the patient's clinician if SPECT was selected as part of the planned investigations had the patient not participated in the trial. Where the SPECT study is performed for study purposes only (and not part of the initial clinical diagnostic pathway) the results may be made available by specific request following the coronary angiogram, to guide patient management for example with regard to identification of inducible ischaemia.

### Investigation Reporting

All test results will be reported by independent clinical and/or research staff, who will be blind to the results of all other investigations.

CMR data sets will be simultaneously analysed by two cardiologists with extensive experience in CMR and the following details will be recorded by consensus:

• Evidence of ischaemia by visual comparison of rest/stress CMR perfusion scans (16 segments of the 17-segment American Heart Association (AHA)/American College of Cardiology (ACC) model, excluding the apical segment). A score of 0 (normal), 1 (equivocal), 2 (subendocardial ischaemia) or 3 (transmural ischaemia) will be assigned to each segment.

• Presence and severity (% luminal narrowing) of coronary artery stenosis on the coronary MR angiogram (17 coronary sections).

• Evidence of scar tissue (17 myocardial segment model). A score of 0 (none), 1 (1–25%), 2 (26–50%), 3 (51–75%) or 4 (>75%) will be allocated to each segment.

• Evidence of regional wall motion abnormalities (17 myocardial segment model). Segmental wall motion will be scored as 0 (normal), 1 (mild hypokinesia), 2 (severe hypokinesia), 3 (akinesia) or 4 (dyskinesia).

• Quantitative analysis will include: end systolic volume (ml), end diastolic volume (ml), ejection fraction (%), left ventricular mass (g), scar tissue mass (g).

The quality of each CMR component will be graded on a scale from 1 (poor) to 4 (excellent). A combined CMR report based all CMR data will report whether the patient has any evidence for CHD and whether this is functionally significant as per the study definition. The affected myocardial territories will be specified. Other cardiac and extracardiac abnormalities will be noted.

Nuclear data sets will be analysed simultaneously by a cardiologist (CD) with extensive experience in nuclear cardiology and an experienced medical physicist. The following details will be recorded by consensus:

• Evidence of ischaemia by visual comparison of rest/stress SPECT perfusion scans (based on the standard 17-segment AHA/ACC model. A score of 0 (normal), 1 (equivocal), 2 (moderately reduced), 3 (severely reduced) or 4 (absent) will be assigned to each segment.

• Evidence of ischaemia by semi-quantitatively scoring using the QPS 20 segment, 5 point model (0 = normal, 1 = mildly reduced uptake, 2 = moderately reduced uptake, 3 = severely reduced uptake and 4 = Absent uptake). Summed stress and rest scores will be based on the 20 segment model.

• Evidence of regional wall motion abnormalities (17 myocardial segment model). Segmental wall motion will be scored as 0 (normal), 1 (mild hypokinesia), 2 (severe hypokinesia), 3 (akinesia) or 4 (dyskinesia).

• Quantitative analysis will include end systolic volume (ml), end diastolic volume (ml), ejection fraction (%).

As for CMR, the study quality will be reported and a summary SPECT report will be produced with an assessment of whether the patient and which vascular territory shows evidence for CHD and whether this is functionally significant as per the study definition.

X-ray angiography images will be analysed by two cardiologists (JY and NM) with experience in invasive coronary angiography. Quantitative coronary angiography (QCA) analysis will be performed off-line using QCAPlus software (Sanders Data Systems, Palo Alto, California, USA) and the following details will be recorded:

∘ Coronary artery dominance

∘ Location and percentage of stenosis by visual estimation and by QCA in each of the main coronary arteries

∘ Vessel diameter

∘ Ventricular function if assessed by left ventriculography

ETT will be reported by the requesting clinician according to the criteria of the ACC/AHA guidelines for exercise testing [21], and the results collected including all the ETT data specified above. In addition the clinician's opinion on the ECG result (positive, borderline positive negative, or equivocal for ischaemia) will be recorded.

### Annual follow-up

Annual follow-up over the subsequent 3 years will be undertaken. A number of procedural safeguards will be followed to establish survival prior to a follow-up telephone call. Information to be obtained at the telephone consultation includes, medical history since randomisation including details and dates of; acute coronary syndrome, emergency or elective revascularisation procedure, any admission for cardiovascular cause including heart failure, cardiac arrhythmia, suspected cardiac event, acute CHD hospitalisation, stroke/transient ischaemic attack (cerebrovascular). These data will be verified from hospital or family practitioner records. Details of any recent cardiovascular investigations will be taken. In addition, Office of National Statistics (ONS) monitoring will be sought for deceased patients to determine the certified causes of death. Notification of deaths by ONS is independent of the patients' clinical follow-up (if they remain resident in the United Kingdom) and ensures recording of unbiased cause-specific mortality both during the study and after, for an indefinite period.

### Definition of outcomes/events

#### Diagnosis of CHD

Significant CHD will be defined as ≥ 70% stenosis of a first order coronary artery measuring ≥ 2 mm in diameter at X-ray coronary angiography, or left main stem stenosis ≥50%.

#### Major adverse coronary events

To assess the prognostic value of CMR, patients will be followed for three years from test completion for the occurrence of a major adverse coronary event (MACE) which will be a composite endpoint comprising at least one of the following; death from cardiovascular cause, acute coronary syndrome (with clinical and biomarker evidence), late revascularisation (>3 months after the initial coronary angiogram) and hospital admission for any cardiovascular cause.

### Statistical considerations

#### Sample size

For our primary objective, estimating the diagnostic accuracy of CMR against the reference standard of X-ray angiography we calculated that a sample size of 750 patients would enable us to estimate the sensitivity or specificity of CMR to within +/-3.5%. This assumes a sensitivity (or specificity) of 90% for CMR and 60% prevalence of significant CHD in the study population and is based on the precision of the 95% confidence interval for a proportion. For the secondary objectives of comparing the diagnostic accuracy of CMR with SPECT and also ETT, this number would be sufficient to detect a 6.5% difference in sensitivities (or specificities) between CMR and SPECT with 80% power, assuming a sensitivity of 85% for SPECT and 2-sided analyses at the 5% significance level [22]. As the sensitivity and specificity of ETT is expected to be lower at around 68% sensitivity and 77% specificity, our sample size will provide sufficient power to detect the expected larger differences between CMR and ETT.

To assess the prognostic value of CMR in predicting MACE approximately 10 events are required for each prognostic factor in the statistical model [23]. The prognostic value of the combined CMR conclusion will be assessed after adjusting for the known risk factors age, gender, cholesterol, blood pressure, smoking and diabetes, which would require 70 events. It is estimated that approximately 30% of patients will undergo planned revascularisation based on the angiogram result and for this to be carried out usually within 8 weeks. For the remaining patients we estimate that for every 70 patients, 10 will be normal with an event rate of <1% per year and 60 will have CHD (30 significant and 30 mild) with an event rate of approximately 5% per year. The required sample size of 750 patients for the diagnostic accuracy evaluation would therefore result in approximately 23 patients experiencing an event per year. With a minimum follow-up period of 3 years this would provide the required number of 70 patients with an event.

#### Analysis plan

Analysis of the primary objective will be completed when every patient has completed all of the diagnostic tests. All statistical testing will be performed at a 2-sided 5% significance level. A final statistical analysis plan will be written and reviewed before any statistical analysis is undertaken.

#### Analysis populations

The analysis population for the primary objective will include all patients who have results for both CMR and X-ray angiography. The analysis populations for the secondary objectives will include all patients who have the appropriate data for each comparison. Patients withdrawing from three year follow-up will be censored at the time of withdrawal.

#### Missing data

The numbers of patients with missing data for one or more tests, and the numbers of uninterpretable tests will be reported. Patients with missing data for one or more diagnostic tests will be excluded from any comparisons involving those tests.

#### Test conduct

The numbers of patients undergoing each test will be reported along with reasons why the test was not performed, where available. The duration between the CMR and X-ray angiography and also between the SPECT and X-ray angiography will be summarised. Details of the order of the testing sequences for each patient will also be summarised.

### Primary objective

#### Diagnostic accuracy – CMR compared to angiography

The sensitivity, specificity, positive and negative predictive values and corresponding 95% confidence intervals for the diagnostic performance of CMR in detecting the presence of significant CHD as confirmed by the X-ray angiogram will be calculated. This will evaluate the combined CMR conclusion. Additional analyses will also be performed to explore the diagnostic accuracy of the individual CMR components: late enhancement, perfusion, contractile function and CMR coronary angiography. These components will be examined alone and in combination (with a positive result for CHD on one or more tests being taken as a positive overall) to identify which component of the CMR data has the best ability to detect CHD.

### Secondary objectives

#### Comparison of CMR with SPECT and ETT

The sensitivity, specificity, positive and negative predictive values and corresponding 95% confidence intervals for the diagnostic performance of SPECT and ETT compared with the X-ray angiogram will be calculated. For the comparison of CMR with SPECT and also with ETT we will use McNemar's test to compare the sensitivities and specificities.

#### Prognostic value of CMR (at 3 year follow-up)

The prognostic ability of the combined CMR result alone and after adjusting for the risk factors: age, gender, cholesterol, blood pressure, smoking and diabetes in predicting time to the first major cardiovascular event will be assessed using Cox proportional hazards modelling. Patients not experiencing an event will be treated as censored at their last known follow-up assessment. Hazard ratios and 95% CI will be reported and Kaplan-Meier curves will be calculated. This analysis will be for all patients. Allowing physicians access to SPECT results is ethically unavoidable but may also influence revascularisation decisions and thus introduce an analysis bias. In order to account for any possible bias and any differences in the risk of future events resulting from decisions about revascularization we will conduct additional analyses which will adjust for the impact of revascularization on outcomes by treating it as an intermediate event, using time-dependent covariates and multistate models[24].

#### Adverse events

All serious adverse events that occur as a result of the diagnostic tests will be reported by diagnostic test. No formal statistical testing will be undertaken.

#### Cost effectiveness

The cost-effectiveness of alternative diagnostic strategies will be evaluated using data from CEMARC and published sources. This will take the form of a decision analytic model, which will provide a framework to link CEMARC data on the diagnostic accuracy of the alternative forms of diagnosis, information on therapeutic implications and evidence on the ultimate costs to the UK National Health Service and health benefits to patients in the form of quality-adjusted life-years (QALYs) [25]. In addition to information on diagnostic accuracy, CEMARC will provide data on clinicians' therapeutic plans following diagnostic information provided by the alternative diagnostic modalities. CEMARC will also provide data on the costs of diagnostic tests. Using evidence from relevant systematic reviews, the effects and costs of those therapies for a given type of patient will be estimated and incorporated into the model. The cost-effectiveness of alternative diagnostic strategies will, therefore, be determined by the extent to which the information they provide results into cost-effective changes in treatments. Modelling methods to reflect long-term implications of changes in therapy for QALYs and costs will reflect recent models published in the cardiovascular area [26-28]

The general methods used will follow those defined as good practice by the National Institute for Health and Clinical Excellence (NICE) [29]. This will include modelling the full range of diagnostic strategies which would be considered in the National Health Service (NHS), using systematically identified estimates for all input parameters, the use of probabilistic sensitivity analysis to express the uncertainty in all elements of evidence in terms of the probability that each diagnostic strategy is the most cost-effectiveness and further scenario analysis to explore the implications of key assumptions made in the model. We will also use value of information analysis to assess where future research in this area is likely to be the most valuable [25].

### Data monitoring

Data will be monitored for completeness and quality by the CTRU. A full monitoring schedule, including Serious Adverse Events and Adverse Reactions, will be established and agreed by the Programme Steering Committee (PSC) and Study Management Group (SMG).

## Discussion

The CE-MARC study is a prospective, diagnostic accuracy cohort study in a population of 750 out-patients presenting with chest pain. It aims to assess the diagnostic accuracy, for detection of significant CHD, using a multi-parametric CMR protocol versus X-ray coronary angiography, nuclear scintigraphy (SPECT) and exercise tolerance testing (where feasible). The prognostic value of CMR and its cost effectiveness will also be compared with these modalities. CE-MARC will be the largest prospective trial to date to compare CMR against standard non-invasive investigations for the diagnosis of CHD, and may have important implications for the wider adoption of CMR in the future.

## Competing interests

The authors declare that they have no competing interests.

## Authors' contributions

JPG, AR, JMB, JN, AJF, CD, JFY, JPR, MS, SP: Participated in the design and co-ordination of the study and helped to draft the manuscript. NM: Participated in the co-ordination of the study and helped to draft the manuscript. SGB: Conceived of the study, participated in the design and co-ordination and helped to draft the manuscript. All authors read and approved the final manuscript.
